# The Promotion of Healthy Hydration Habits through Educational Robotics in University Students

**DOI:** 10.3390/healthcare11152160

**Published:** 2023-07-29

**Authors:** Alejandro De la Hoz, Lina Melo, Andrés Álvarez, Florentina Cañada, Javier Cubero

**Affiliations:** Health Education Laboratory, Department of Experimental Science and Mathematical Education, University of Extremadura, 06006 Badajoz, Spain; alexdlhoz@unex.es (A.D.l.H.); lvmelo@unex.es (L.M.); andalvarez@unex.es (A.Á.); flori@unex.es (F.C.)

**Keywords:** hydration, robotic educational, obesity, university student, digital health education

## Abstract

In recent years, there has been a lack of healthy lifestyle habits in the population, including hydration, with negative consequences for health. At the same time, advances in technology have changed the process of teaching and learning since elementary school, highlighting the incorporation of educational robots as innovative resources in recent years. This study analyzes the state of the scientific knowledge presented by university students doing a university degree in Primary Education after a robotics-based educational intervention. The study adopted a quasi-experimental design with a qualitative approach, using category systems and a quantitative approach with descriptive and inferential (Chi-square and Contingency Coefficient) statistics. The results of the study show that the level of scientific knowledge has improved in the different scientific contents involved, highlighting the excellent level presented for the recommended daily volume of hydration. Innovative interventions, through digital resources such as Educational Robotics, are presented as possible alternatives to promoting the healthy habit of hydration, due the effective learning of biosanitary knowledge in the young population.

## 1. Introduction

Childhood overweight and obesity continue to increase. In the particular case of Spain, according to the data from the latest Nutritional Study of the Spanish Population (ENPE), almost 40% of children between 3 and 8 years of age suffer from these problems. The figures are not much more encouraging if adolescents and young people are also considered. Thirty-four percent of the population between 3 and 24 years of age suffer from overweight or obesity, with the majority being boys: in them, this prevalence rises to 39.2%, while in girls it is 28.4% [1]. Within this problem, one of the main risk factors is the excessive intake of sugary drinks, to the detriment of the recommended healthy consumption of water. Therefore, one of the main focuses of public health should be to implement interventions that promote the hydration habits of the young general population [2]. These prevalence data may have been aggravated as a consequence of the COVID-19 pandemic and confinement, assumed as the main factors of a sedentary lifestyle and inadequate diet, mainly through the inadequate intake of sugary beverages and soft drinks, as a consequence of the lack of education on healthy eating habits [3].

On the other hand, our current society is constantly changing, and one of the most important changes is rapid digitalization. Students in a globalized world based on science and technology require scientific training in health-related topics, as well as an adequate digital literacy to enable them to acquire the digital skills necessary for processing all the information that they are faced with every day [4,5]. This can be observed by internet searches on health content, which are performed using terminology with worrying deficiencies [6], leading to misconceptions due to a lack of scientific and digital competency. Under this paradigm, proper digital literacy is necessary, and especially digital health literacy, which is understood as the skills and competency of an individual to access, understand, and use health-related information on the internet [3,7].

The level of literacy of a person is linked to the scientific knowledge he/she possesses on health issues, thus having an impact on the acquisition of healthy lifestyle habits that allow for a healthy lifestyle to be achieved. Accordingly, a person with a low level of literacy is more likely to have health-related problems [8], so it is necessary to obtain an adequate level of literacy in the population. Previous studies have shown that active interventions [9,10] based on digital resources [3,11] allow for improving these levels of digital literacy, as well as their scientific knowledge and self-efficacy in health-related content.

In addition to this, there is global concern about the state of the health of the population, due to the fact that, in recent decades, there has been an increase in the number of people with health problems and the diseases associated with them, as a result of unhealthy lifestyle habits [11,12]. In this sense, the hydration statuses of people become especially important, especially in a society marked with an excessive consumption of sugary and energy drinks. Therefore, the population, especially children and young people, should learn the adequate scientific knowledge to carry out healthy hydration habits [13,14,15,16,17]. It is important to highlight the importance of water, which is essential in the human body, being the main component of our organism. It is considered to be a nutrient by the European Food Safety Authority (EFSA) [18] and constitutes an essential biomolecule for all vital functions. Its metabolism is regulated through its intake in liquid form through beverages or in the form of food, supplementing its production with metabolic water, while its loss is mainly through urine, feces, skin, lungs, and sweat [13].

Adequate hydration is associated with an improved cognitive performance in terms of concentration [19], physical performance in hot environments [20], the prevention of kidney stone formation [21], and other health benefits [14,15]. Healthy hydration is associated with optimal cognitive performance, while reduced hydration significantly affects this [13]. A study conducted on schoolchildren, in which their hydration status was assessed via urine osmolality, showed an association between dehydration and decreased short-term memory and verbal analogy. Likewise, previous studies have shown inadequate water intake in both adult, child, and youth populations [22].

Thus, compliance with dietary intake recommendations is related to compliance with the Adequate Intake (AI) of EFSA [18]. Similarly, the correct consumption of other types of beverages that complement hydration is necessary, in which a distinction is made between beverages such as natural juices, milk, and dairy products, which should be consumed daily, and other sugary and/or carbonated beverages, which should only be consumed occasionally [14,15,16]. Nissensohn [23] exposed the need to address the EFSA recommendations on the development of methodologies for the evidence-based appropriate learning of fluid intake. 

Despite this, there is little scientific literature that focuses exclusively on hydration, and it has mostly focused on the hospital setting. Previous studies have demonstrated that appropriate population-based intervention allows for improvements in the scientific knowledge about hydration [24,25,26]. In this context, Health Education (HE) is an essential tool in the Health Promotion for Public Health, within numerous Areas of Knowledge, among which, the framework of didactics in Experimental Sciences stands out, where it has a greater relevance, given its high educational value. The objective is not only for the population to acquire healthy lifestyle habits that allow them to achieve a healthy lifestyle, but also to obtain the development of competency and capabilities in terms of bioscientific knowledge about health content, so that they can be applied in preventive and health-promoting decision making [27]. 

The school is presented as a primordial scenario in health promotion, so that teachers should integrate health interventions in their didactic programs. This is why future teachers need adequate training in and updating on scientific knowledge and digital literacy [28,29]. However, studies [9] have shown that there are training gaps in the curricula on health education content. Likewise, it has been shown that teachers in training do not present adequate scientific knowledge, as well as adequate digital literacy, since their training is limited to knowledge related to the human body, its functioning, and its relationship with the environment and hygiene, so progress in intervention programs is necessary [27]. 

However, it has been shown that appropriate interventions in the university context, and specifically for future teachers, allow for improving these levels [3,10,27]. Thus, HE must reverse this situation through didactic strategies that enable meaningful learning in students, in line with also achieving the Sustainable Development Goals (SDGs). In this particular case, HE is aligned with several Sustainable Development Goals, such as 3: Health and Well-being, 4: Quality Education, and 6: Clean Water and Sanitation [30]. Under the magnifying glass of the scientific and technological society in which we find ourselves, the use of innovative digital tools, as well as the use of cooperative active methodologies, are fundamental for the achievement of these objectives (UNESCO) [31]. A previous study [11] analyzed the differences in scientific knowledge about hydration after an intervention based on a technological application, and its results showed that this type of digital resource is effective for the improvement of scientific knowledge about hydration. 

As a result of this evolution, the paradigm shift is linked to the use of digital tools such as robotics or programming [32,33]. Educational Robotics (ER) is presented as a set of actions for the creation and use of robotics projects. In recent years, ER has increased its inclusion in classrooms and in the scientific literature as a consequence of the benefits it presents. Several studies have demonstrated its positive impact on the development of skills such as computational thinking, critical thinking, problem solving, creativity, motivation, and metacognitive skills [34,35,36,37,38]. According to the scientific literature, learning via robotics means that students learn knowledge from different areas, such as science and mathematics, and biosanitary content through the use of robots [38,39,40,41,42]. Despite this, there is very little scientific literature analyzing the influence of ER on improving curricular content, especially bioscience content, and existing studies usually focus on improving the knowledge and skills related to the robot itself and its handling [40,41,42,43]. Benitti [44] and Ferrada [45] identified studies that presented the advantages of the use of ER in teaching content, including scientific content, at the Early Childhood Education stage [46], in Primary Education [47], or in Secondary Education [48]. Despite the limited scientific literature on the use of ER in the university context, Schina et al. [49] and Román et al. [50] studied the use of robotics in the teaching of scientific content and sustainable development goals, identifying an improvement in the understanding and practical application of this content. 

In view of the above, the innovative resource of Educational Robotics is presented as a possible ally in the promotion of healthy lifestyle habits such as hydration from the infant to juvenile population, and particularly in university students doing a degree in Education. For this reason, the aim of this study is to analyze the degree of improvement in the bioscience knowledge level about the healthy habit of hydration, through an intervention based on Educational Robotics.

## 2. Materials and Methods

### 2.1. Study and Participants

The study adopted a quasi-experimental design with a mixed qualitative research analysis methodology (QUAL-cuan) [51]. A qualitative approach via the analysis of category systems was complemented with a quantitative analysis using descriptive and inferential statistics [52,53]. 

The design and implementation of the didactic proposal was carried out through convenience sampling with a total of 116 students (78 females and 38 males) from the University of Extremadura. Specifically, it was performed in practical seminars in the Di-dactics of the Mathematics I subject. This course corresponded to the third year of the academic year, so the average age of the students was 21.77 (±1.18). The students had received a basic introduction to ER as a resource for enhancing logical mathematical learning in their previous year of studies. This study was performed in line with the principles of the Declaration of Helsinki [54].

### 2.2. Intervention

The intervention was based on a learning process based on Educational Robotics, with the aim of promoting the healthy habit of hydration, through the learning of the most relevant biosanitary concepts of this habit, such as the recommended number of glasses, the total volume, and the recommended and non-recommended drinks, etc. The aim of the intervention was to increase the students’ scientific knowledge, as well as their knowledge of the innovative digital resources applicable to the teaching of bioscientific content.

Specifically, this intervention was under a Challenge-Based Learning methodology, a methodology used in previous studies for the use of ER [55]. The objective was the creation, through the formation of collaborative groups of 4 students, of robotic boards for teaching the healthy habit of hydration. In addition, the students were required to produce reports documenting the process of creating and explaining the created board. These reports would include images, descriptions, and other necessary resources to provide guidance on how to use the robotic board in an educational classroom. The creation of the robotic boards and their corresponding reports only entailed the mandatory use of different bioscience concepts relevant to the teaching of healthy hydration habits, allowing for the development of skills such as creation and problem solving for the university students.

The intervention was carried out in 3 practical sessions of one hour, complemented with non-presential work for the creation of the proposed activity. It began with a first active session on the practical management of the possibilities presented by the Mind Designer^®^, where the students themselves began to manage the robot through its programming in the App. The Mind Designer App^®^ presents a simple and intuitive interface that allows a simple programming sequence for learning the basic notions of programming. At the same time, the App presents variables of this robotics kit that allow for the creation of geometric figures in a simple way, while allowing the drawing of them in a physical way on a sheet of paper.

At the same time, the functionality of the robotic boards was explained to them with the use of this robotics kit. For this purpose, robotic boards on bioscientific content were used [31], allowing the students to understand and reflect on the variety of options that can be employed for use in a classroom. The amount of resources offered by Mind Designer^®^ characterizes it as being appropriate for teaching bioscience content to students at the elementary school level.

This intervention continued with two more practical sessions to carry out the pro-posed project. In these sessions, the students were guided in the search, analysis, and use of biosanitary content, through specialized health websites or platforms, such as the WHO or MedlinePlus, and databases such as PubMed or ERIC. It should be made clear that all the websites had to have the Digital Health Quality Seal: Health On Net Foundation (HON), which ensures their scientific and biosanitary rigor, providing the students with resources to improve their Digital Health Literacy [3].

### 2.3. Measures and Data Analysis

In order to determine the level of the scientific knowledge of the university teachers in training, a mixed research analysis methodology (*QUAL-cuan*) [51] with a system of categories was created (Table 1) to analyze the different robotic boards on healthy hydration, as well as the associated reports documenting the process of creation and explanation. For the creation of the system of categories, the Grounded Theory technique was used, which consists of the formation of the system of categories from the sources of the information received, with the aim of completing pre-established systems [52,53]. In addition, attention was paid to the most relevant content that the students should know in order to acquire adequate learning of the healthy hydration content, according to previous studies and the recommendations of international entities [14,15,16,17,18]. In this regard, it was analyzed that the biohealthy content not only appeared on the board, but that the report included a scientific justification of the need to work on the specific content. For example, the recommended number of glasses, which drinks are more or less healthy, and the reasons for this, etc., should be described.

In this way, the frequency and percentage of occurrence of the different categories and subcategories on the boards and reports were analyzed, in which an adequate knowledge of this specific content was demonstrated. This was only used at the end (post-test) of the intervention because, following a previous generic oral screening, it was observed that the participants had no previous scientific knowledge. To determine this level of scientific knowledge, a scale of categories (Table 2) created in a previous study [3] was used. The scale was created according to the percentage of correct answers given by the students in a questionnaire on bioscientific content. In the present study, the scale was used based on the percentage of occurrences in each category and subcategory over the total number of projects analyzed.

For the data analysis, the qualitative analysis software “ATLAS.ti” [56] was used. This is a software that allows for the organization, analysis, and interpretation of qualitative information, which presents resources such as the creation of coding systems, memos, and networks. In the case of the inferential analysis, the ‘R-Commander’ interface from “R” was used to perform the different statistical tests [57]. The Chi-square statistical test was performed to analyze the level of relationship between the 4 categories used.

## 3. Result

**Figure 1:**
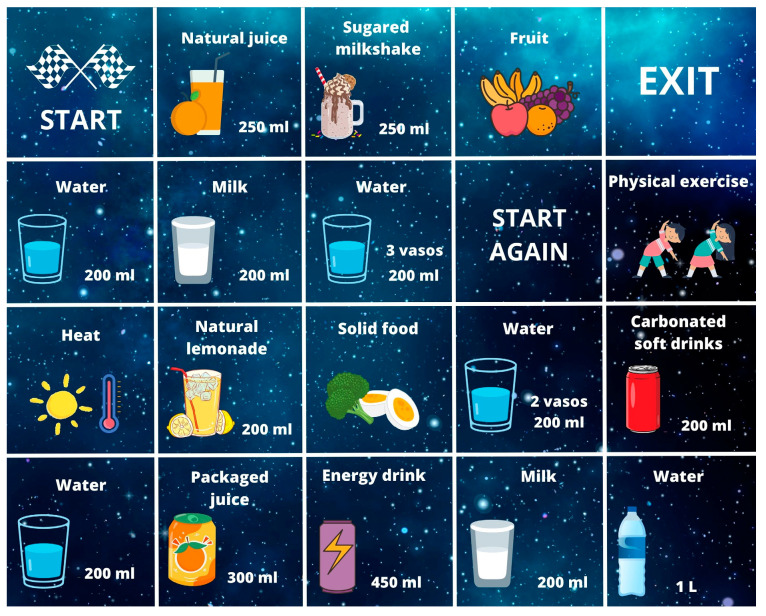
Example of a Robotic board for Healthy Hydration. Elaboration by university students.

Figure 1 shows an example of the design and creation of the robotic board that the university teachers in training had to create. It shows the use of the different scientific content they had to learn, such as the volume of glasses (200 mL) and the incorporation of more and less healthy drinks (natural juices and packaged juices, etc.).

In addition to Figure 1, Appendix A shows an example of the associated report de-scribing, detailing, and explaining the robotic board, how it was used, and the content that was worked on, so that it can be seen that there was a scientific justification for each piece of content to be included in the board as an important part of the healthy habit of hydration.

Appendix A shows some of these appreciations, for example, “*We should drink 2 L of water a day, that is, 10 glasses. However, we can substitute glasses of water for another beverage that is for daily consumption, being 6 glasses the minimum of water*”. Another example is the following: “*The protagonists will have to look for strategies... on the condition that every day they drink ten glasses of 200 mL each, of which at least six must be water, the rest will have to be combined according to the requirements of the Healthy Hydration Pyramid*”.

It can be observed that the general volume of water that is recommended was shown, as well as its relationship with the corresponding number of glasses, and the minimum number of glasses that should be ingested. The knowledge of the measure of the glasses with which the recommendations of scientific rigor are carried out, such as the Pyramid of Healthy Hydration, is also observed.

Table 3 shows the results obtained from the qualitative analysis of the robotics boards and their associated reports. The frequency of the coding of each subcategory can be observed, as well as the corresponding percentage it represents over the total number of boards and reports (n = 29). In addition, the level of scientific knowledge in which each subcategory is present can be observed as a function of the percentage acquired [3].

The subcategory with the highest percentage of occurrence is *Milk*, with 89%, followed by the subcategories *2 L*, *200–250 mL,* and *Natural juice* with 76%. On the other hand, the subcategories with the lowest percentage of occurrence are *Natural lemonade* with 17%, *Energy drinks* with 21%, and *6 glasses* with 35%.

If we look at the scale in Table 2, which allows us to determine the scientific knowledge about the habit of hydration according to the percentage, we can see that there are four subcategories with an *Excellent* level, (*2 L, 200–250 mililiters, Milk,* and *Natural juice*), four with a *Sufficient* level (*10 glasses, Coffee, tea and infusions, Sweetened soft drinks,* and *Sugared juice/milkshake*), two with a *Problematic* level (*6 glasses* and *Gazpachos*), and two with *Inadequate* level (*Natural lemonade* and *Energy drinks*). Table 4 shows the average percentage of each category, according to the percentages of the subcategories belonging to each one of them.

It is possible to appreciate the *Hydration volume* category with an *Excellent* level (76%), the *Healthy drinks* category with a *Sufficient* level (56%), and the two categories of *Number of glasses* and *Unhealthy drinks* with a *Problematic* level (45% and 48%).

Referring to the inferential analysis, Table 5 shows the results of the *p*-value Chi-square χ^2^ statistical test and the Contingency Coefficient (C) that were performed across the four categories.

The results show the existence of a statistically significant association (*p* < 0.05) of the category of *Number of glasses* with *Healthy drinks* (0.03) with a low strength of association (0.38), and with *Unhealthy drinks* (0.00), with a medium strength of association (0.50). In addition, there is also a statistically significant association between *Healthy Drinks* and *Unhealthy Drinks* (0.00), with a medium strength of association (0.50). Regarding the *Hydration volume* category, this does not present a statistically significant association with any of the remaining categories.

## 4. Discussion

Obesity is a worldwide public health problem associated with an increased risk of cardiovascular and renal diseases, type 2 diabetes mellitus, and problems related to body image. In Spain, according to the latest and worrying data from the ENPE Study, almost 40% of children between 3 and 8 years of age suffer from these problems. This is progressively increasing in the adolescent and juvenile population. Finally, 34% of the population between 3 and 24 years of age suffer from overweight or obesity [1]. 

Thus, a sedentary lifestyle and inadequate diet, mainly occurring through the inadequate intake of sugary beverages and soft drinks, have been described as triggers of the problems of overweight and obesity, as a consequence of the lack of basic knowledge on nutrition and education on healthy eating habits [2]. For all these reasons, the recommendations for beverage intake to acquire the habit of healthy hydration should be based on adequate scientific knowledge on nutrition [24,25]. This fact becomes more important for active teachers and teachers in training, since they must transmit and teach scientific knowledge to their students, who usually do not have very healthy hydration habits, as well as a poor knowledge about this healthy habit [26]. 

The present study aimed to determine the level of scientific knowledge that university students doing a degree in Education acquired after an intervention based on the use of Educational Robotics, also offering them an experience that allowed them to acquire digital competence in this tool for teaching and literacy in scientific content on nutrition. 

For this purpose, a mixed methodology was used, in which the main use of the qualitative approach was employed, which allowed for providing non-pre-established data and obtaining results through an in-depth analysis of the content; at the same time, it was complemented with the use of a quantitative approach to give mathematical value with a statistical analysis [51,52,53].

The results of the study show that the teachers in training increased their level of scientific knowledge after the intervention, in the same way that has occurred in previous studies [3,10,11,12,27], which carried out active interventions and were based mainly on technology and digital literacy for health.

Starting from non-existent or low knowledge, it can be observed that the level of scientific knowledge on some content was *Excellent* (Table 4), as was the case of *Hydration Volume*. Knowledge of this content is fundamental, since previous studies [23,26,58] have demonstrated the existence of a low fluid intake, especially if we consider the hydration volume of water, an essential nutrient in our body.

If we look at *healthy drinks*, the level of scientific knowledge was *Sufficient*. In this category, it should be noted that there were some subcategories that did present a specific level of scientific knowledge of *Excellent*, such as *Milk* and *Natural juices*, which are considered to be the main beverages consumed by children and young people after water [14,15,18]. Accordingly, it should be noted that the teachers in training belonged to the primary education stage, so that the level of knowledge analyzed in these two beverages should be highlighted due to their relevance to the age of the targeted students.

On the other hand, there were two categories with a *Problematic* level, the *Number of glasses* and *Unhealthy drinks*. In this case, if we analyze the subcategories of *Unhealthy Drinks*, we observe that the percentage of *Energy Drinks* (21%) decreased the average percentage notably. It should be noted that the use of this type of beverage is not very frequent in the children and youth populations, so that its low frequency of appearance may be due to this circumstance. On the other hand, the other two subcategories, *sweetened soft drinks* and *Sugared juice/milkshake*, did show higher percentages, especially *sweetened soft drinks*. This fact is in line with previous reports and studies that have reported the great current problem of a high consumption of these beverages in the population [17,58]. Therefore, as was the case with *Healthy Drinks*, even though the overall scientific knowledge level on *Unhealthy Drinks* was not as high, the results were positive, as they addressed the beverages that need more attention on the children and youth populations.

Regarding the *number of glasses*, there was a poorer scientific understanding between the minimum number of glasses of water (6) and the adequate number of glasses that a person should consume to hydrate properly each day (10). These results may be due to the fact that some quality resources used to work with hydration content, such as the Healthy Hydration Pyramid [16], make more reference to the adequate amount of consumption and not so much to the minimum amount. In this way, more emphasis is placed on the total recommended intake volume than on the minimum, so it is necessary to place more emphasis on this content in subsequent studies.

The results of the present study show that, in general, there was an acceptable biosanitary knowledge of the students after the intervention. These results coincide with previous studies [44,45,46,47,48], which have reflected an improvement in scientific knowledge after the implementation of interventions based on the use of Educational Robotics, especially in undergraduates doing education degrees [49,50], which have an impact on the inclusion of this resource as being suitable for any educational stage for the formation of scientific knowledge. At the same time, it allows teachers in training to acquire knowledge on the use of ER for teaching content at the primary education stage, a key moment for teaching content related to health and the formation of healthy lifestyle habits. Therefore, the introduction of these digital resources should be increased in didactic programs.

The Challenge-Based Learning (CBL) methodology in the inclusion of Educational Robotics [55] is an alternative that previous studies have considered to be adequate. In view of our positive results, it is considered that this line should be followed in subsequent studies. However, as mentioned above, the results are analyzed in detail after the intervention, as a consequence of the lack of previous knowledge in studies. This means that it was not possible to perform an inferential analysis of the data before and after the intervention, which is a factor to be taken into account in order to improve future interventions.

On the other hand, if we look at the results of the statistical tests (Table 5), we observe that there was a statistically significant (*p* < 0.05) association between the categories of *Number of glasses, Healthy drinks*, and *Unhealthy drinks*, with the *Volume of hydration* category being the only one that did not present a significant association with any of the others. This coincides with the results in Table 4, where the resulting percentages were similar except in the *Hydration volume* category, which presented a higher percentage than the rest.

Although the strength of association between the categories that presented a statistically significant association was medium or low, the results of the statistical test show a certain level of relationship between the learning of certain content, as was the case between *Healthy Drinks* and *Unhealthy Drinks*. This coincides with previous studies [23] that have highlighted the need for the comprehensive learning of the different content that completes the healthy hydration habit, since, on many occasions, these studies have not integrated the evaluation of the different beverages. In this sense, attention is usually paid to sugar-sweetened beverages (mostly in studies conducted on children and adolescents) and alcoholic beverages (in studies on adults). Therefore, we should not only focus on the intake of the healthiest beverages, but learning should also be directed at learning about the intake of unhealthy beverages, thus allowing us to acquire significant learning about the differences between the intake of these different beverages.

The same occurred between the *Number of glasses* and *healthy* and *unhealthy drinks*, in which there was a significant association. This relationship stemmed from the connection established during the learning between the consumption of beverages and the number of glasses to be consumed, especially with the *unhealthy drinks*, since the guideline of consuming a glass infrequently and on a weekly basis is usually set. Therefore, as mentioned above, the learning of hydration content should be performed in a comprehensive manner and not only attend to the consumption of water and its adequate intake, but with emphasis placed on the global computation.

In view of these results, the intervention based on Educational Robotics seems to be an adequate resource in the fight against the worrying public health situation in which we live. The results of the present study show that, in general, there was an acceptable bio-health knowledge of the students after the intervention, and especially for concepts such as the amount of milliliters of the glasses, the general volume of hydration that should be ingested daily, and the knowledge of daily and non-daily drinks. At the same time, it is advocated to increase the use of mixed approaches that allow for exploiting the advantages and strengths of each approach. In addition, it is important to consider that this program allows for the promotion several Sustainable Development Goals, such as 3: Health and Well-being, 4: Quality Education, and 6: Clean Water and Sanitation [30].

## 5. Conclusions

The alarming increase in health problems such as diabetes and obesity in young people, together with the need to achieve the Sustainable Development Goals (SDGs), re-quires Public Health interventions for the promotion of healthy lifestyles, including healthy hydration habits. 

Under the new educational digital paradigm, Educational Robotics is presented as an innovative digital resource that allows for the development of digital literacy and the learning of biosanitary content; therefore, its implementation in higher education training curricula is encouraged.

## 6. Study Limitations and Future Lines of Research

In terms of the study’s limitations, there are some aspects that need to be addressed in future research that were not analyzed in the present study. One of the important aspects is to extend the study sample, as well as to have a scenario with balanced samples in terms of gender variables. Moreover, in this way, it would be convenient to analyze the levels of biosanitary knowledge in an individual manner, with respect to the sex variable as an indicator of the possible differences that should be considered in the analysis of the data obtained.

Another limitation was the duration of the intervention and the study. It would be recommended to increase the time of the intervention, as well as to add control groups to allow for the analysis of possible differences. It should also be extended to longitudinal studies in order to provide a better generalization of the results obtained. A future line of research to complement this type of study would be to analyze how behavior is modified through detailed dietary records, depending on whether or not the knowledge acquired is improved.

## Figures and Tables

**Table 1:** Category system for the analysis of the scientific knowledge of hydration.

Scientific Knowledge	Main Dimension	Categories	Subcategories
Scientific Knowledge Hydration	Water	Number of glasses	10 glasses
			6 glasses
		Hydration volume	2 L
			200–250 mL
	Other drinks	Healthy drinks	Milk
			Natural juice
			Tea and infusions
			Gazpachos
			Lemonade natural
		Unhealthy drinks	Sweetened soft drinks
			Sugared juice/milkshake
			Energy drinks

**Table 2:** Qualitative categories of the scientific knowledge [3].

Categories	Percentages
*Inadequate*	0–25%
*Problematic*	26–50%
*Sufficient*	51–75%
*Excellent*	76–100%

**Table 3:** Frequency, percentage, and level of scientific knowledge of the subcategories (n = 29).

Main Dimension	Categories	Subcategories	Frequency	Percentage	Scientific Knowledge Level
Water	Number of glasses	10 glasses	16	55%	Sufficient
		6 glasses	10	35%	Problematic
	Hydration volume	2 L	22	76%	Excellent
		200–250 mL	22	76%	Excellent
Other drinks	Healthy drinks	Milk	26	89%	Excellent
		Natural juice	22	76%	Excellent
		Coffee, tea and infusions	17	59%	Sufficient
		Gazpachos	12	41%	Problematic
		Natural lemonade	5	17%	Inadequate
	Unhealthy drinks	Sweetened soft drinks	20	69%	Sufficient
		Sugared juice/milkshake	16	41%	Problematic
		Energy drinks	6	21%	Inadequate

**Table 4:** Average percentages and level of scientific knowledge of the categories (n = 29).

Main Dimension	Categories	Frequency	Percentage Average	Scientific Knowledge Level
Water	Number of glasses	13	45%	Problematic
	Hydration volume	22	76%	Excellent
Other drinks	Healthy drinks	16	56%	Sufficient
	Unhealthy drinks	14	48%	Problematic

**Table 5:** Chi-square test χ^2^ (*p*-value) and Contingency Coefficient (C) between categories.

Categories	Chi-Square Test χ^2^ (*p* < 0.05)	Contingency Coefficient (C)
Number of glasses—Hydration volume	0.14	0.26
Number of glasses—Healthy drinks	0.03 *	0.38
Number of glasses—Unhealthy drinks	0.00 *	0.50
Hydration volume—Healthy drinks	0.46	0.14
Hydration volume—Unhealthy drinks	0.18	0.24
Healthy drinks—Unhealthy drinks	0.00 *	0.50

* Statistically significant differences (*p* < 0.05).

## Data Availability

For our study could put in contact with all authors.

## Appendix A

Example of a report for the use of Robotic board for promotion of Healthy Hydration.

## References

[1] Pérez-Rodrigo C., Bárbara G.H., Citores M.G., Aranceta-Bartrina J. (2022). Prevalencia de obesidad y factores de riesgo cardiovascular asociados en la población general española: Estudio ENPE. Rev. Esp. Cardiol..

[2] Salas-Salvadó J., Maraver F., Rodríguez-Mañas L., Sáenz de Pipaon M., Vitoria I., Moreno L.A. (2020). Importancia del consumo de agua en la salud y la prevención de la enfermedad: Situación actual. Nutr. Hosp..

[3] De la Hoz A., Cubero J., Melo L., Durán-Vinagre M.A., Sánchez S. (2021). Analysis of digital literacy in health through active university teaching. Int. J. Environ. Res. Public Health.

[4] Im H., Swan L.E. (2019). Qualitative exploration of critical health literacy among Afghan and Congolese refugees resettled in the USA. Health Educ. J..

[5] Thapa-Bajgain K., Bajgain B.B., Dahal R., Adhikari K., Chowdhury N., Chowdhury M.Z., Turin T.C. (2023). Health literacy among members of the Nepalese immigrant population in Canada. Health. Educ. J..

[6] Hernández-Rabanal C., Vall A., Boter C. (2018). Formación, la clave para mejorar las competencias informacionales en e-salud del alumnado de bachillerato. Gac. Sanit..

[7] Mávita-Corral C.J. (2018). Alfabetización en salud de una comunidad universitaria del noroeste de México en el año 2016. Investig. Educ. Médica.

[8] Davó M.A., Vives-Cases C., Benavides F.G., Alvarez-Dardet C., Segura-Benedicto A., Icart T., Bosch F. (2011). Common public health competencies and content in undergraduate college programs. Gac. Sanit..

[9] Charro-Huerga E., Charro M.E. (2017). Formación del profesor de primaria en educación para la salud. Didact. Exp. Soc. Sci..

[10] Cubero J., Sánchez S., Vallejo J.R., Luengo M.L., Calderón M., Bermejo M.L. (2018). Aprendizaje cooperativo para la formación universitaria en alfabetización en salud. FEM Rev. Fund. Educ. Médica.

[11] Steven A., Wilson G., Young-Murphy L. (2019). The implementation of an innovative hydration monitoring APP in care home settings: A qualitative study. JMIR Mhealth Uhealth.

[12] Cubero J., Gracía M., Sánchez S., Vallejo J.R., Rodrigo M., Ramírez-Moreno J.M. Análisis de Alfabetización en Salud en Estudiantes Universitarios Socio/Sanitarios y Usuarios de Oficina de Farmacia. Proceedings of the 8º Congreso Ibero-Americano em Investigação Qualitativa 2019 (CIAIQ2019).

[13] Perales-García A., Ortega R.M., Urrialde R., López-Sobaler A.M. (2018). Evaluación del consumo de bebidas, ingesta dietética de agua y adecuación a las recomendaciones de un colectivo de escolares españoles de 7 a 12 años. Nutr. Hosp..

[14] Aranceta J., Arija V., Maíz E., Martínez E., Ortega R., Pérez-Rodrigo C., Quiles J., Serra L. (2016). Guías alimentarias para la población española (SENC, diciembre 2016); la nueva pirámide de la alimentación saludable. Nutr. Hosp..

[15] Aranceta J., Gil Á., Marcos A., Pérez-Rodrigo C., Serra-Majem L., Varela-Moreiras G. (2016). Conclusions of the II International and IV Spanish Hydration Congress. Toledo, Spain, 2nd-4th December, 2015. Nutr. Hosp..

[16] Martínez J.R., Iglesias C. (2006). El Libro Blanco de la Hidratación.

[17] Poulos N.S., Pasch K.E. (2016). Socio-demographic differences in energy drink consumption and reasons for consumption among US college students. Health Educ. J..

[18] EFSA Panel on Dietetic Products, Nutrition, and Allergies (NDA) (2010). Scientific opinion on dietary reference values for fats, including saturated fatty acids, polyunsaturated fatty acids, monounsaturated fatty acids, trans fatty acids, and cholesterol. EFSA J..

[19] Khan N.A., Raine L.B., Drollette E.S., Scudder M.R., Cohen N.J., Kramer A.F., Hillman C.H. (2015). The relationship between total water intake and cognitive control among prepubertal children. Ann. Nutr. Metab..

[20] Wilk B., Timmons B.W., Bar-Or O. (2010). Voluntary fluid intake, hydration status, and aerobic performance of adolescent athletes in the heat. Appl. Physiol. Nutr. Metab..

[21] Prezioso D., Strazzullo P., Lotti T., Bianchi G., Borghi L., Caione P., Zattoni F. (2015). Dietary treatment of urinary risk factors for renal stone formation. A review of CLU Working Group. Arch. Ital. Urol. Androl..

[22] Fadda R., Rapinett G., Grathwohl D., Parisi M., Fanari R., Calò C.M., Schmitt J. (2012). Effects of drinking supplementary water at school on cognitive performance in children. Appetite.

[23] Nissensohn M., López-Ufano M., Castro-Quezada I., Serra-Majem L. (2021). Valoración de la ingesta de bebidas y del estado de hidratación. Rev. Esp. Nutr. Comunitaria..

[24] Benelam B., Wyness L. (2010). Hydration and health: A review. Nutr. Bull..

[25] Chen S.H., Huang Y.P., Shao J.H. (2017). Effects of a dietary self-management programme for community-dwelling older adults: A quasi-experimental design. Scand. J. Caring Sci..

[26] De la Hoz A., Sánchez S., Vega M.R., Benavente M.J., Cubero J. (2020). Analysis of hydration habit and its knowledge in a sample of 10–12 year-old schoolchildren in the province of Badajoz (Spain). Rev. Esp. Nutr. Comunitaria..

[27] Franco-Reynolds L., De la Hoz A., Valles C., Benavente M.J., Sánchez S., Cubero J. (2022). Efecto sobre el hábito de la dieta mediterránea de una intervención cooperativa en estudiantes Universitarios. Rev. Esp. Nutr. Comunitaria..

[28] Nguyen A., Eschiti V., Bui T.C., Nagykaldi Z., Dwyer K. (2023). Mobile Health Interventions to Improve Health Behaviors and Healthcare Services among Vietnamese Individuals: A Systematic Review. Healthcare.

[29] Chai H.H., Gao S.S., Chen K.J., Duangthip D., Lo E.C.M., Chu C.H. (2020). A Kindergarten-Based Oral Health Preventive Approach for Hong Kong Preschool Children. Healthcare.

[30] Gil C.G. (2018). Objetivos de Desarrollo Sostenible (ODS): Una revisión crítica. Papeles Relac. Ecosociales Y Cambio Glob..

[31] UNESCO Tecnologías Digitales al Servicio de la Calidad Educativa. Una Propuesta de Cambio Centrada en el Aprendizaje Para Todos. https://unesdoc.unesco.org/ark:/48223/pf0000245115.

[32] De la Hoz A., Cañada F., Melo L.V., Álvarez A., Cubero J. Design of a robotic board for teaching the water cycle. Proceedings of the 14th International Conference on Education and New Learning Technologies (EDULEARN22).

[33] De la Hoz A., Cañada F., Melo L.V., Álvarez A., Cubero J. Teaching human hydration science content through computational thinking and educational robotics. Proceedings of the 15th Conference of European Science Education Research Association (ESERA).

[34] Bers M.U. (2017). The Seymour test: Powerful ideas in early childhood education. Int. J. Child—Comput. Interact..

[35] Chiang F.K., Zhang Y., Zhu D., Shang X., Jiang Z. (2022). The Influence of Online STEM Education Camps on Students’ Self-Efficacy, Computational Thinking, and Task Value. J. Sci. Educ. Technol..

[36] Sun Y., Chang C.H., Chiang F.K. (2022). When Life Science Meets Educational Robotics. Educ. Technol. Soc..

[37] Tselegkaridis S., Sapounidis T. (2022). Exploring the Features of Educational Robotics and STEM Research in Primary Education: A Systematic Literature Review. Educ. Sci..

[38] Espinosa-Garamendi E., Labra-Ruiz N.A., Naranjo L., Chávez-Mejía C.A., Valenzuela-Alarcón E., Mendoza-Torreblanca J.G. (2022). Habilitation of Executive Functions in Pediatric Congenital Heart Disease Patients through LEGO^®^-Based Therapy: A Quasi-Experimental Study. Healthcare.

[39] Schina D., Valls C., Borrull A., Usart M., Esteve-González V. (2021). An associational study: Preschool teachers’ acceptance and self-efficacy towards Educational Robotics in a pre-service teacher training program. Int. J. Educ. Technol. High. Educ..

[40] Chang C.Y., Hwang G.J., Chou Y.L., Xu Z.Y., Jen H.J. (2023). Effects of robot-assisted digital storytelling on hospitalized children’s communication during the COVID-19 pandemic. Educ. Tech. Res. Dev..

[41] Marcos-Pablos S., García-Peñalvo F.J. (2022). More than surgical tools: A systematic review of robots as didactic tools for the education of professionals in health sciences. Adv. Health Sci. Educ..

[42] Smakman M.H., Konijn E.A., Vogt P.A. (2022). Do Robotic Tutors Compromise the Social-Emotional Development of Children?. Front. Robot. AI.

[43] Belmonte J.L., Sánchez S.P., Bújez M.R.V., Mohedo M.T.D. (2019). Herramientas robóticas para la dinamización de nuevos espacios educativos. Campus Virtuales.

[44] Benitti F.B.V. (2012). Exploring the educational potential of robotics in schools: A systematic review. Comput. Educ..

[45] Ferrada C., Carrillo-Rosúa F.J., Díaz-Levicoy D., Silva-Díaz F. (2020). La robótica desde las áreas STEM en Educación Primaria: Una revisión sistemática. Educ. Knowl. Soc. (EKS).

[46] Sánchez M.E., Gutiérrez R.C., Somoza J.A.G.C. (2019). Robótica en la enseñanza de conocimiento e interacción con el entorno. Una investigación formativa en educación infantil. Rev. Interuniv. Form. Profr. RIFOP.

[47] Ntourou V., Kalogiannakis M., Psycharis S. (2021). A study of the impact of Arduino and Visual Programming In self-efficacy, motivation, computational thinking and 5th grade students’ perceptions on electricity. Eurasia J. Math. Sci. Technol. Educ..

[48] Fussero G.B., Occelli M. (2022). Construcción de modelos de Ingeniería Genética a través de la programación con Scratch. Eureka J. Sci. Teach. Dissem..

[49] Schina D., Esteve-González V., Usart M., Lázaro-Cantabrana J.L., Gisbert M. (2020). The integration of sustainable development goals in educational robotics: A teacher education experience. Sustainability.

[50] Román-Graván P., Hervás-Gómez C., Martín-Padilla A.H., Fernández-Márquez E. (2020). Perceptions about the use of educational robotics in the initial training of future teachers: A study on steam sustainability among female teachers. Sustainability.

[51] Creswell J.W. (2014). A Concise Introduction to Mixed Methods Research.

[52] Vives V.T., Hamui S.L. (2021). La codificación y categorización en la teoría fundamentada, un método para el análisis de los datos cualitativos. Investig. Educ. Med..

[53] Amezcua M., Gálvez-Toro A. (2002). Los modos de análisis en investigación cualitativa en salud: Perspectiva crítica y reflexiones en voz alta. Rev. Esp. Salud Publica.

[54] World Medical Association (WMA) Declaration of Helsinki—Ethical Principles for Medical Research Involving Human Subjects. https://www.wma.net/policies-post/wma-declaration-of-helsinki-ethical-principles-for-medical-research-involving-human-subjects/.

[55] Conde M.Á., Rodríguez-Sedano F.J., Fernández-Llamas C., Gonçalves J., Lima J., García-Peñalvo F.J. (2021). Fostering STEAM through Challenge-Based Learning, Robotics, and Physical Devices: A Systematic Mapping Literature Review. Comput. Appl. Eng. Educ..

[56] Cipollone M.D. (2022). Atlas.ti como recurso metodológico en investigación educativa. Anu. Digit. Investig. Educ..

[57] Ocaña R. (2019). Descubriendo R-Commander.

[58] Mayo M.A.B., Izquierdo J.Q. (2019). Ingesta de líquidos e hidratación en personas mayores no institucionalizadas en un municipio de Valencia (España). Rev. Esp. Nutr. Comunitaria.

